# Supplementary material for: A homology-guided, genome-based proteome for improved proteomics in the alloploid Nicotiana benthamiana
Source: BMC Genomics. 2019 Oct 4;20:722. doi: 10.1186/s12864-019-6058-6 (PMC6778390; doi:10.1186/s12864-019-6058-6)
Supplement: Supplementary file 1 — Figure S1. Comparison of Solanaceae proteomes. Figure S2. Phylogenetic analysis of the subtilase gene-family with names. Figure S3. Phylogenetic analysis of the subtilase gene-family of tomato and Arabidopsis and including other previously characterized subtilases. (PDF 518 kb) [file 12864_2019_6058_MOESM1_ESM.pdf]

Supplemental figures Kourelis et al: *A homology-guided, genome-based proteome for improved proteomics in the alloploid Nicotiana benthamiana*

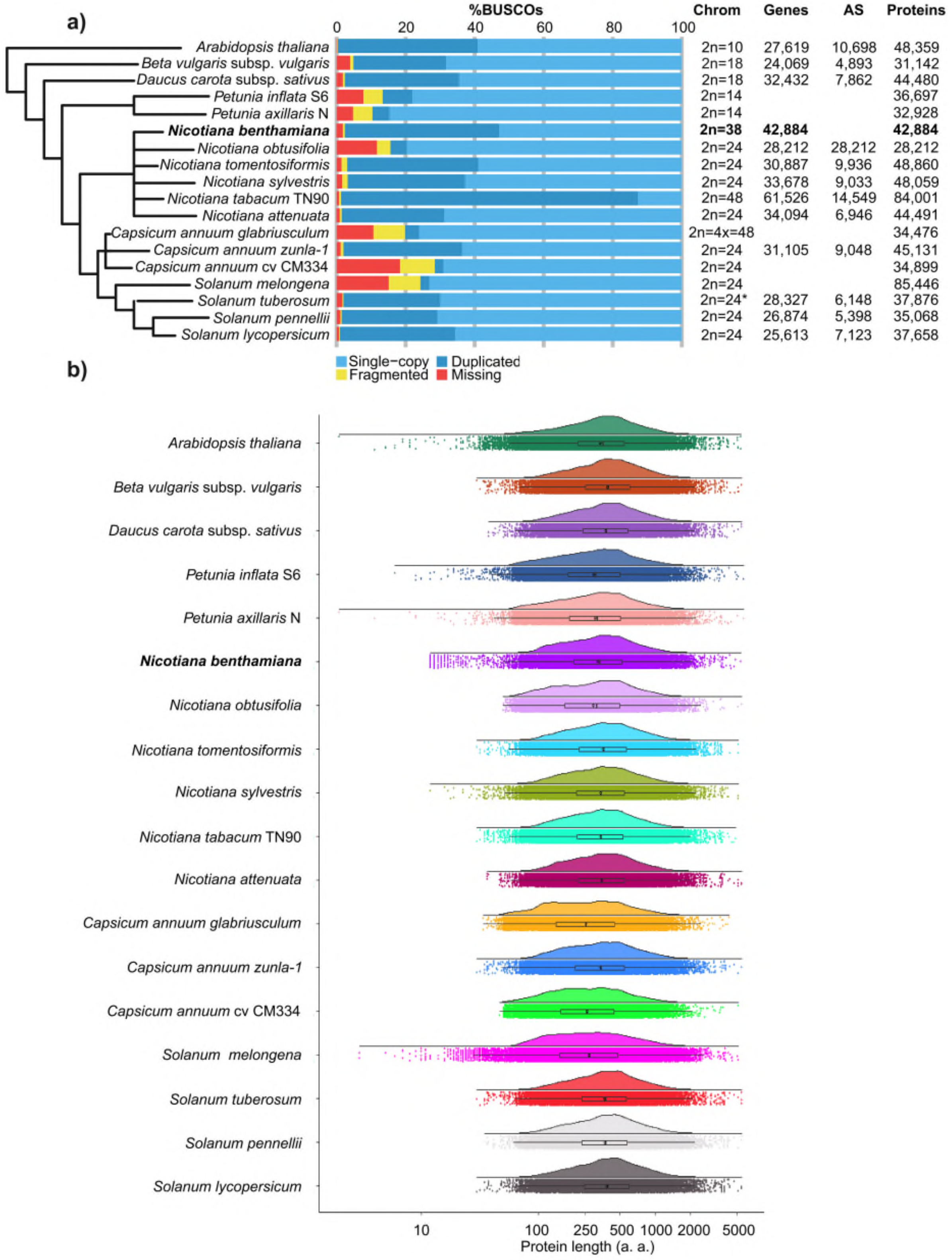

Figure S1: Comparison of Solanaceae proteomes. **a)** Completeness of the predicted proteomes from sequenced Solanaceae genomes was estimated using BUSCO v3 with the embryophyta database. *Certain *Solanum tuberosum* species are polyploid. **b)** Violin and boxplot g of $\log_{10}$ protein length distribution of each predicted proteome. Jittered dots show the raw underlying data.

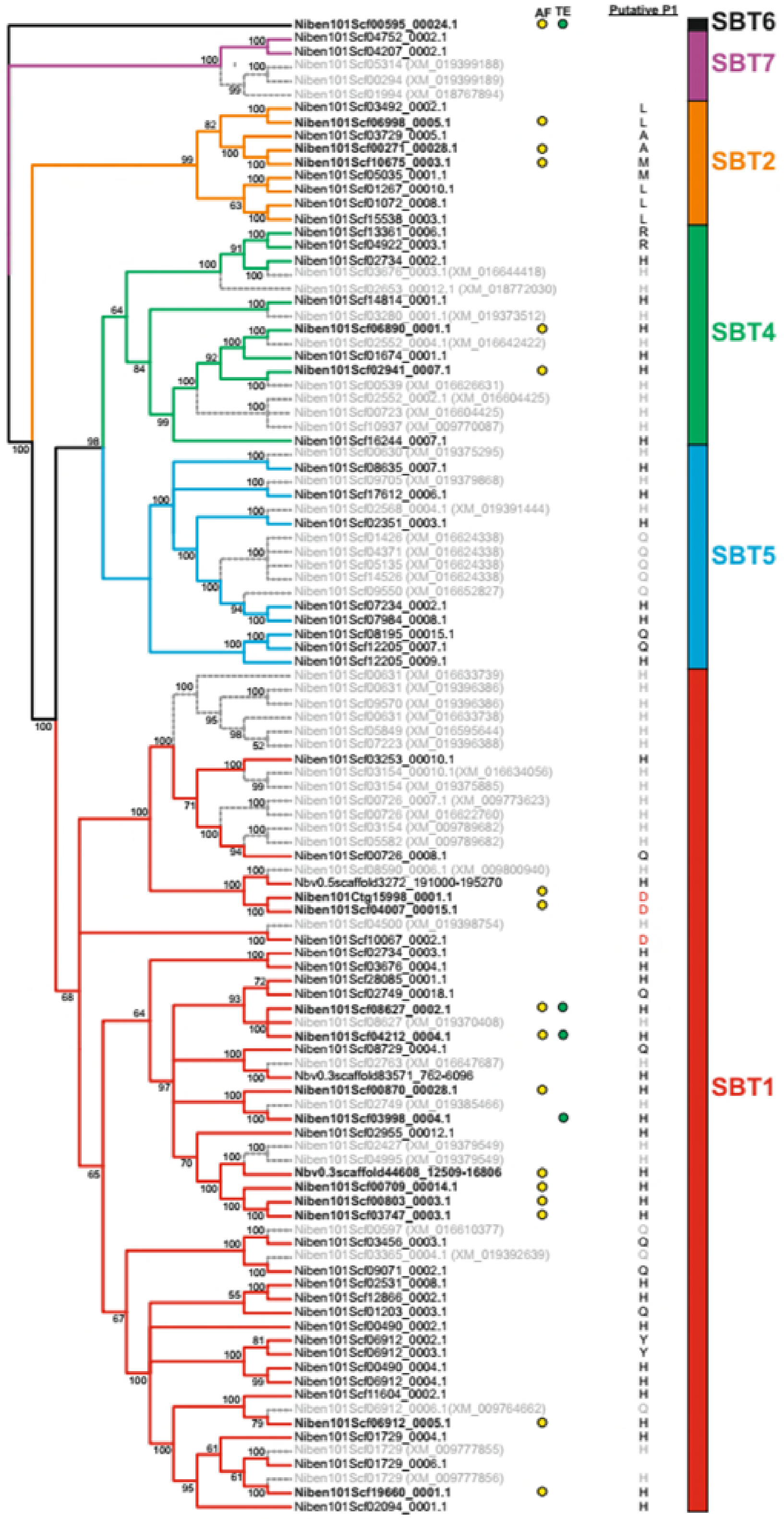

Figure S2: Phylogenetic analysis of the subtilisin gene-family with full names. The evolutionary history of the subtilase gene family was inferred by using the Maximum Likelihood method based on the Whelan and Goldman model. The bootstrap consensus tree inferred from 500 replicates is taken to represent the evolutionary history of the taxa analysed. Putative pseudogenes are indicated in grey. Subtilases identified in apoplastic fluid (AF) and/or total extract (TE) are indicated with yellow and green dots, respectively. Putative P1 based on residue at the prodomain junction is indicated, putative phytaspases are indicated in red. Naming of subtilase clades according to (Taylor and Qiu, 2017).

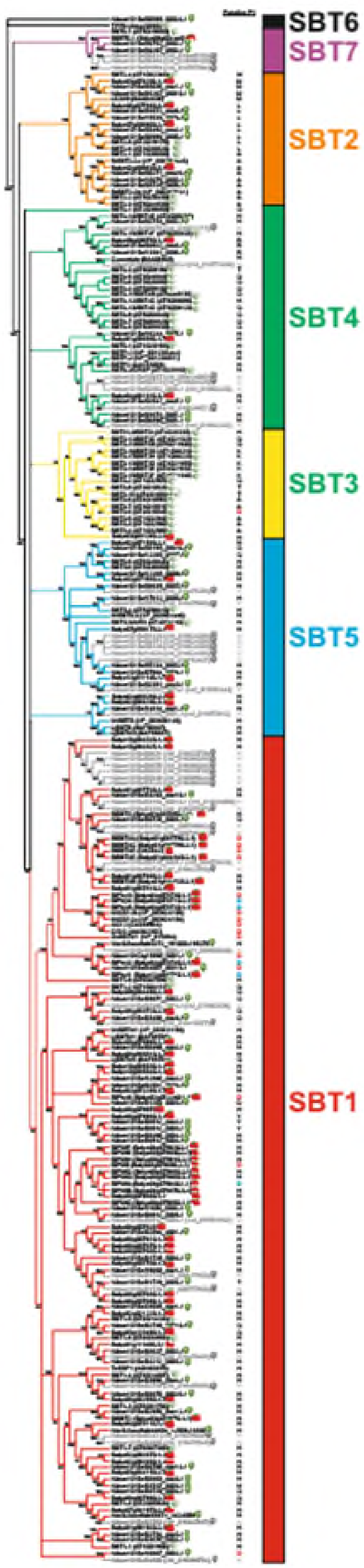

Figure S3: Phylogenetic analysis of the subtilisin gene-family of tomato and Arabidopsis and including other previously characterized subtilisins. The evolutionary history of the subtilase gene family was inferred by using the Maximum Likelihood method based on the Whelan and Goldman model. The bootstrap consensus tree inferred from 250 replicates is taken to represent the evolutionary history of the taxa analysed. Putative pseudogenes are indicated in grey. Putative P1 based on residue at the prodomain junction is indicated. Putative phytaspases are indicated in red, confirmed phytaspases in blue. Naming of subtilase clades according to (Taylor and Qiu, 2017).
